# Interdisciplinary Case Study: Understanding the Cooperation of Humans and Robots through the Collaboration of Social and Computer Scientists

**DOI:** 10.1016/j.isci.2020.101680

**Published:** 2020-11-03

**Authors:** Hirokazu Shirado, Nicholas A. Christakis

**Affiliations:** 1 School of Computer Science, Carnegie Mellon University, Pittsburgh, PA 15213, USA; 2 Yale Institute for Network Science, Yale University, New Haven, CT 06520, USA; 3 Department of Sociology, Yale University, New Haven, CT 06520, USA; 4 Department of Ecology & Evolutionary Biology, Yale University, New Haven, CT 06511, USA; 5 Department of Biomedical Engineering, Yale University, New Haven, CT 06520, USA

**Figure:**
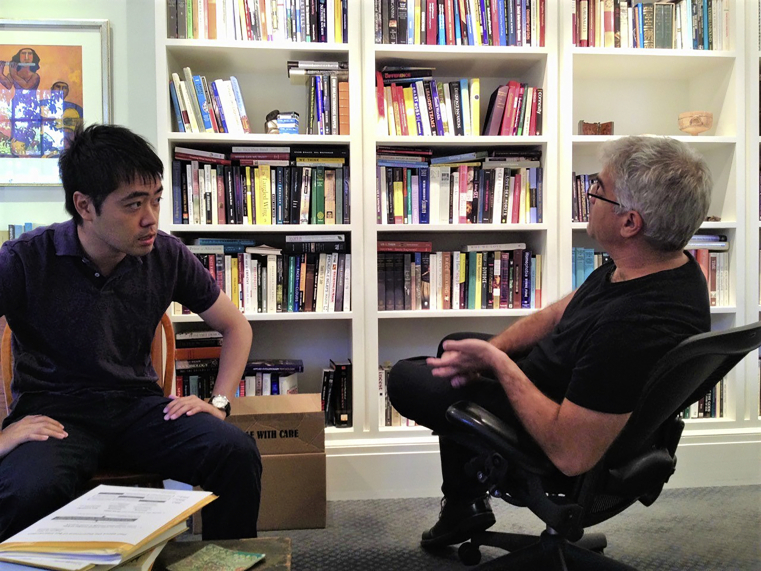
Hirokazu Shirado (Left) and Nicholas A. Christakis (Right) Sit Together Discussing Their Interdisciplinary Research Early on in Their Collaboration

In our research, we have been obsessed with the idea that it should be possible to use artificial intelligence (AI), to support not only individual humans, such as physicians making difficult decisions or athletes perfecting their game, but also groups of humans trying to work together better. Can AI be used to help humans to help themselves? So, for over 5 years, we have been collaborating on a deepening set of projects with this objective. The article published recently in *iScience*, “Network Engineering Using Autonomous Agents Increases Cooperation in Human Groups” (https://www.cell.com/iscience/fulltext/S2589-0042(20)30630-1#%20) represents a culmination, in some ways, of this effort—because, among other things, we are able to show that certain kinds of “simple AI,” when introduced into human groups, can actually enhance levels of cooperation in human groups.

Cooperation is challenging because it creates a social dilemma: the group does best if people cooperate, but each individual is always templated to keep their resources given their self-interest. It is also clear that individual people, if they do not have some overarching power (like being the “boss”), can find it very difficult to facilitate group cooperation. Hence, in our view, as the authors of this work, new, decentralized approaches to the challenge of cooperation would be very helpful—especially if they could involve the deployment of AI. How might individuals we can call “reformers”—and, in particular, bots (i.e., AI agents)—embedded within a group and simply acting locally facilitate cooperation in a wider population?

Using online experiments with hundreds of people, we find that bots can improve (and not just maintain) cooperation in human groups when the bots are allowed to adjust social ties locally and engage in a kind of “social network engineering” to address the challenges faced by reformers attempting to make the world better. In this work, we presented what is, to our knowledge, the first intervention in human groups that can actually increase, and not merely preserve, levels of cooperation found at baseline. Deploying these decentralized, simply programmed AI agents helped. Ironically, cooperation between several disciplines was needed to come to such a conclusion.

## Proximity

### Who Were the Players in This Project, and How Did You Bring Everyone Together?

This project was done by Hirokazu Shirado (Assistant Professor; the School of Computer Science at Carnegie Mellon University, http://www.shirado.net) and by Nicholas A. Christakis (Professor; Yale Institute for Network Science, with appointments in the Departments of Sociology, Medicine, and Data Science). Hirokazu was a former graduate student of Nicholas, and we worked together on the project in Human Nature Lab (Nicholas's lab, www.humannaturelab.net). We also had technical support for the requisite software for our experiments (available at breadboard.yale.edu) by software engineers in the lab, principally Mark McKnight, and Hirokazu's specifications about what the software needed to be able to do helped the software engineers to perfect their product, too.

For one, each of us has made efforts to learn other disciplines.

## Language

### Did You Encounter Any Challenges or Any Benefits of Working with People from Different Backgrounds and Expertise?

We have benefitted a lot from our interdisciplinary collaboration. Hirokazu brings engineering ideas and technological skills to the challenge of designing and performing the online social experiments. Nicholas brings theoretical and analytical frames in social sciences and biology to make our work more meaningful and relevant to the workings of our society. Together, we are interested in what we have come to call “hybrid systems” of humans and machines. We have not encountered difficulties from this interdisciplinary collaboration. For one, each of us has made efforts to learn other disciplines. Hirokazu, who was a robotic engineer, completed a PhD program in Sociology. Nicholas, who was a medical doctor and is a professor in sociology, has many collaborations, including with engineering and computer science researchers. Moreover, we work in the same field of network science and computational social science, which is also inherently interdisciplinary. And our familiarity with collaborative inter-disciplinary projects helped us to collaborate smoothly in this project.

## Research Methods

### Did This Project Require Tailoring Your Research Methods to Adjust to Working Interdisciplinarity?

We did not need to tailor our research methods for this project, in part because we have jointly developed them. Since we started to work together in 2011, we had tested several methods to support our work, including developing the requisite software (“Breadboard,” available at breadboard.yale.edu); procedures for recruiting online research subjects (over the years, many thousands of people have participated); and fundamental emulations of social systems (for instance, in this experiment in *iScience*, we make use of group dynamics we had tested and published in the past).

## Governance

### How Did the Decision of Branching Out from Your Fields Come About? Are There Funding Challenges?

The interdisciplinary field bridging the social/behavioral sciences and computational/engineering sciences is getting more and more essential, in our view, given the impact of technology on our society, and in particular the transition from the analog age to the digital age. Thus, our decision to work in this interdisciplinary field felt natural to us. As noted above, we are deeply interested in the topic of “hybrid systems” of humans and machines. For an example of careers, after first training in robotics in Japan, and then earning a PhD in Sociology in the USA, Hirokazu got his first job as an Assistant Professor in the School of Computer Science at Carnegie-Mellon, in part because they judged his interdisciplinary work as pertinent and exciting. Funding for interdisciplinary research can be a struggle, but we have been able to do it, with this work being funded by the Robert Wood Johnson Foundation and the NOMIS Foundation.

This [publishing or presenting in interdisciplinary venues] could lead (and previously has led) to future collaborations to explore new frontiers of science.

## Publication

### When Publishing This or Any Interdisciplinary Paper, How Do You Decide Which Community/Venue to Target?

We like to have our work published by interdisciplinary journals because we can communicate with experts in a wide range of disciplines. This could lead (and previously has led) to future collaborations to explore new frontiers of science (for instance, we have begun to think about the application of some of our ideas to the topic of animal migration and movement, in part stimulated by the work of Iain Couzin and others, and to the topic of “machine behavior” more generally, in part stimulated by the work of Iyad Rahwan and others). Workshops or small conferences led by publishers or funders to discuss a specific interdisciplinary topic might help open-minded researchers to find new collaborations, in our view.

## Final Thoughts

### What Did You Learn About Interdisciplinary Research from the Project and What Tips Would You Give to Anyone Considering Undertaking Such Work?

Honestly, we have greatly enjoyed the whole research process involved in this project. We believed that we would gain new insights with respect to the cooperation and coordination of humans and AI/machines in hybrid systems because our interdisciplinary collaboration was so unusual. We think we have succeeded in gaining some new insights. A key success factor, for us, might be that we have the same fundamental interests in social networks and in the challenges of collective action. We naturally respect each other's knowledge, expertise, and ideas to approach these scientific challenges together.

